# Supplementary material for: The causal relationship between the human gut microbiota and pyogenic arthritis: a Mendelian randomization study
Source: Front Cell Infect Microbiol. 2024 Nov 26;14:1452480. doi: 10.3389/fcimb.2024.1452480 (PMC11629706; doi:10.3389/fcimb.2024.1452480)
Supplement: Supplementary file 1 [file DataSheet1.zip › mendelian test/ebi-a-GCST90027690.csv_leave_one_out.pdf]

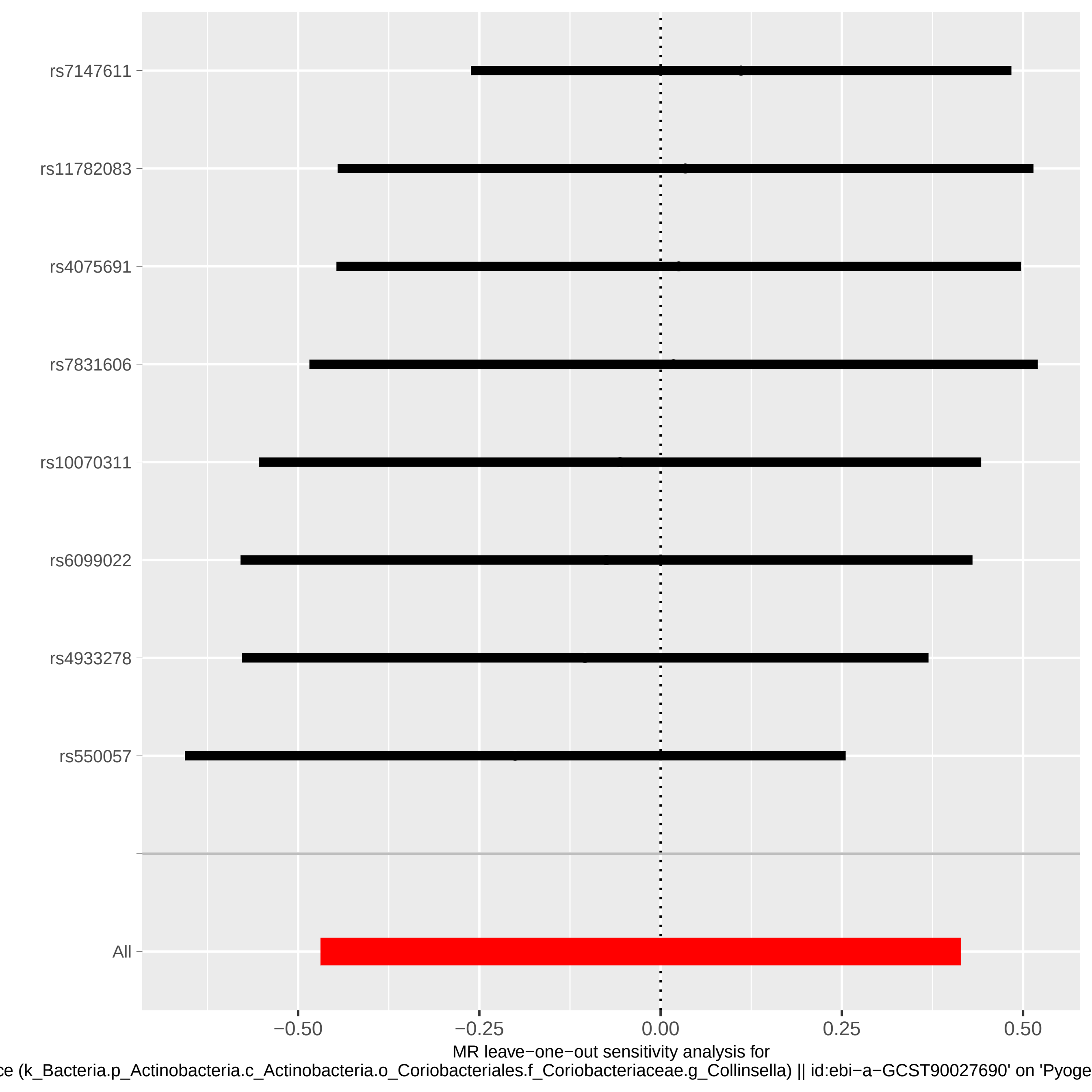

rs7147611
rs11782083
rs4075691
rs7831606
rs10070311
rs6099022
rs4933278
rs550057
All
−0.50
−0.25
0.00
0.25
0.50
MR leave−one−out sensitivity analysis for
ce (k_Bacteria.p_Actinobacteria.c_Actinobacteria.o_Coriobacteriales.f_Coriobacteriaceae.g_Collinsella) || id:ebi−a−GCST90027690' on 'Pyoge
